# Insights from 25 Years of Measles and Measles–Rubella Vaccination Campaigns in the WHO African Region (2001–2025)

**DOI:** 10.3390/vaccines14060549

**Published:** 2026-06-22

**Authors:** Balcha Girma Masresha, Goitom Gebremedhin Weldegebriel, Emmaculate Jepkorir Lebo, Sarah Wanyoike, Ado Mpia Bwaka, Yolande Vuo-Masembe

**Affiliations:** 1 World Health Organization Regional Office for Africa, Brazzaville P.O. Box 06, Congo; 2 World Health Organization Inter-Country Support Team for Eastern and Southern Africa, Harare P.O. Box CY 348, Zimbabwe; weldegebrielg@who.int (G.G.W.); swanyoike@who.int (S.W.); 3 Independent Consultant; emma.lebo@ifrc.org; 4 World Health Organization Inter-Country Support Team for Western Africa, Ouagadougou P.O. Box 7019, Burkina Faso; bwakaa@who.int; 5 World Health Organization Inter-Country Support Team for Central Africa, Libreville, Gabon; masembey@who.int

**Keywords:** African Region, measles, rubella, supplemental immunization activities, mass campaigns, elimination

## Abstract

Introduction: The WHO African Region is working to eliminate measles and rubella in 80% of countries by 2030. In countries with sub-optimal routine immunization coverage, periodic supplemental Immunization Activities (SIAs) are implemented to boost childhood immunity against measles and rubella. Methods: We reviewed the SIA technical reports and reports from post-campaign surveys shared by countries with the WHO Regional Office for Africa, and we analyzed the coverage data from preventive measles campaigns implemented during the years 2001–2025. Results: A total of 326 preventive measles/measles–rubella SIAs were implemented across 44 countries in the years 2001–2025, providing more than 1.5 billion doses of vaccine to eligible children according to the type and scale of the campaigns. Four fifths (82%) of the SIAs were nationwide exercises, and all of the SIAs were implemented as non-selective vaccination campaigns targeting all eligible children irrespective of past vaccination history, with the exception of four SIAs. The 95% administrative SIA coverage target at national level was met in 209 SIAs (64.7%). At district level, 11 of 164 SIAs had 100% of districts attaining 95% administrative coverage. Only 94 SIAs (29%) were followed by post-campaign coverage survey, and only 18 (19%) of these attained coverage of 95% or more by survey. Nearly two thirds (62%) of the 272 SIAs implemented during 2006–2025 had at least one additional intervention included with the measles/MR vaccination. Discussion: Measles and MR vaccination campaigns have served as excellent opportunities for providing integrated child survival interventions in the African Region. While two thirds of the SIAs met the national administrative coverage target, district-level coverage targets were not met in the majority of the SIAs, and only one fifth of the SIAs met the national-level survey coverage targets. Moreover, discrepancies were noted between administrative and survey coverage results, possibly due to inaccuracies in the reporting of the number of doses administered and/or reliance on inaccurate denominators. For optimal impact, SIAs need to adequately reach unreached populations. Conclusions: In view of the documented sub-optimal coverage, countries should provide strong leadership and ownership of the measles elimination strategies for the attainment of the SIA coverage targets as well as the overall measles and rubella elimination goal. There is an urgent need for improved tools to identify unvaccinated children, high-risk populations, and innovative strategies to reach them. All countries implementing SIAs should also include systematic monitoring of zero-dose children, and conduct post-campaign coverage surveys in a timely manner.

## 1. Introduction

Countries in the WHO African Region have been implementing measles control strategies since the year 2000 [1]. Currently, the Regional goal is to eliminate measles and rubella in 80% of countries by 2030 [2]. According to the WHO UNICEF vaccination coverage estimates, the African Regional vaccination coverage with the first dose of measles-containing vaccine (MCV1) increased from 50% in the year 2000 to 71% in 2024, with a third of the countries in the Region having MCV1 coverage estimates of less than 70%, and only five countries attained 95% MCV1 coverage. The Regional coverage with the second dose of measles-containing vaccine (MCV2) in 2024 was 55% [3]. By the end of 2025, the second dose of measles vaccine was provided in 45 of the 47 countries in the Region, while rubella vaccine had been introduced in the routine immunization schedule in 35 countries.

Between 2000 and 2024, countries made substantial progress in reducing measles and rubella disease burden [4,5]. During this period, it is estimated that 21 million measles deaths were averted in the African Region as a result of the implementation of the measles elimination strategies [4]. By the end of 2025, three countries in the Region had attained the verification of measles and rubella elimination [6].

In the presence of sub-optimal routine immunization coverage, many countries continue to depend on mass vaccination campaigns, also referred to as supplemental Immunization Activities (SIAs), to boost childhood immunity against measles and rubella. SIAs are implemented with a view to vaccinate everyone in the target age group within a defined geographic area irrespective of prior vaccination status and prior illness status. The principal objective of SIAs is to ensure to reach previously unvaccinated children (also referred to as “zero-dose children” who have missed the opportunity to get routine doses of measles containing vaccine for any reason, often related to lack of geographic access to or poor utilization of vaccination services). The preparation to implement SIAs often takes more than 6 months and includes mobilizing funds from the global donors and local sources, galvanizing political leadership at all levels, implementing district-level microplanning, intensive advocacy and community mobilization, complex arrangements for transport as well as cold chain and vaccine logistics, and the training and deployment of large numbers of health workers in the weeks preceding and during the service delivery period, which commonly lasts 7–10 days. The implementation of measles/measles–rubella SIAs creates unique opportunities for immunization program managers from the national and provincial levels to reach remote communities and come into contact with the health workforce at the operational level. Hence, measles/measles–rubella vaccination campaigns have been used as platforms for the co-delivery of other child survival interventions, which are integrated as part of the initial planning and intense preparations.

Health workers document information related to the SIA coverage at the site of vaccination service delivery, including the number of vaccine doses provided, the age group of the vaccinated child, and whether the child had received prior routine doses of the measles/MR vaccine or not (zero dose for measles/MR vaccine). This information, initially collected on tally sheets, is aggregated at different administrative levels up to the national level. WHO guidance outlines ≥ 95% coverage at national level and in every district as a benchmark for high-quality measles SIAs [7].

In this manuscript, we attempt to describe and summarize the results of measles and measles–rubella SIAs across the African Region over the last 25 years.

## 2. Methods

We analyzed the coverage data from preventive measles campaigns implemented during the years 2001–2025. Catch-up campaigns (to kickstart measles control or to introduce rubella vaccine) are typically implemented to reach children between the ages of 9 months and 15 years, while periodic follow-up SIAs typically cover children from 9 months up to 5 years of age. Depending on the epidemiological context and the availability of resources, countries may decide to start targeting children from 6 months of age and may change the upper limit of the target age group [7,8].

The SIA technical reports and reports from post-campaign surveys are shared with the WHO Regional Office for Africa, which maintains a database that summarizes the results from every SIA. The database includes information such as the year of implementation, the type and scope of SIAs, the target age group, the additional interventions co-administered in the SIAs, the administrative coverage, the proportion of districts that have attained the target of 95% coverage, and, whenever available, the national-level survey coverage during the SIAs [9]. We reviewed available post-campaign technical reports and post-campaign coverage survey reports. For this analysis, we excluded all vaccination campaigns conducted in response to measles outbreaks. Descriptive statistical analyses were conducted to summarize campaign characteristics, administrative coverage, district performance indicators, and post-campaign survey coverage.

Administrative coverage: Whenever measles or measles–rubella (MR) SIAs are implemented, teams of health workers providing the supplemental vaccination services at fixed, temporary or mobile service sites capture essential service data regarding the vaccine doses provided. This data is tallied and summarized by health service delivery point, district and province, and is finally summarized into a national-level coverage against estimated target population, typically derived from national population projections for the SIA exercise. At the end of the campaign, national immunization programs develop technical reports that include the national and subnational breakdown of administrative coverage as well as additional details on the key lessons learnt and challenges faced.

Post-campaign coverage surveys: Within a few weeks following the implementation of SIAs, countries are expected to organize standard post-campaign coverage surveys (PCCS) in representative samples of the population. The purpose is to generate national- and first-subnational-level estimates of the SIAs’ coverage [7].

## 3. Results

### 3.1. Scope and Scale of SIAs

A total of 326 preventive measles/measles–rubella SIAs across 44 countries are documented in the Regional dataset, which covers the years 2001–2025. A cumulative total of 1,553,231,684 doses of measles/MR vaccines were provided during these SIAs. Three countries—Algeria, Seychelles, and Mauritius—did not report any preventive SIAs during the study period (Table 1). The majority (255 of 326) of the SIAs were nationwide (82%). Thirty-three of the SIAs were MR catch-up campaigns for introducing rubella vaccine, where a total of 195.3 million persons were reached in nationwide campaigns in 31 countries since 2013, as well as 75.7 million children in subnational measles–rubella catch-up campaigns in Nigeria and DR Congo in 2025.

All of the SIAs were implemented as non-selective vaccination campaigns (targeting all eligible children irrespective of past vaccination history) with the exception of four SIAs. These include Senegal which, in 2021, implemented selective SIAs targeting children who were not vaccinated in the past, while Kenya (2021), Eritrea (2024) and Rwanda (2024) tailored their SIAs to target only geographic areas and communities that were considered at high risk.

A total of 207 of the 242 follow-up SIAs (85%) targeted children from 6 or 9 months to 5 years of age. Only 13 follow-up SIAs additionally targeted children in the age bracket up to 10 years and 11 covered children up to 15 years, while one follow-up SIA targeted children up to 7 years of age. Ten SIAs (4%) done between 2010 and 2012 targeted children only up to 4 years of age.

### 3.2. SIA Coverage

**Figure 1:**
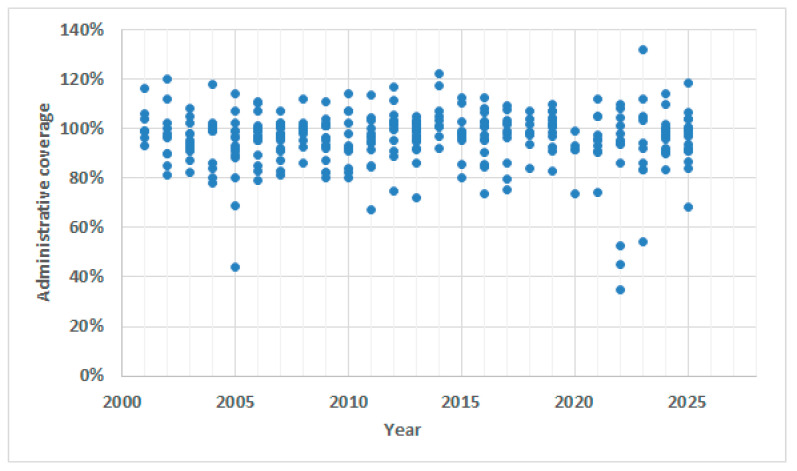
Scatter plot of administrative coverage from measles/MR SIAs in the African Region, 2001–2025. Each dot represents the administrative coverage value of a campaign against the year of implementation.

Data on administrative coverage was not provided for three SIAs; Senegal in 2010, Equatorial Guinea in 2011 and South Africa in 2017. Administrative SIA coverage results of 95% or more at national level were documented in 209 of 323 SIAs (64.7%), while 54 SIAs (16.7%) had coverage of 90–94.9%; 43 SIAs (13.3%) had 80–89.9% coverage, and 17 SIAs (5.3%) scored less than 80% administrative coverage. Reported administrative coverage was more than 100% in 107 (33%) campaigns, with 21 campaigns reporting more than 110% coverage. Very low SIA administrative coverage of <60% was documented from Equatorial Guinea in 2005; from Gabon, Lesotho, Gambia in 2022; and from South Africa in 2023 (Figure 1). Three of the SIA technical reports did not have complete information on the administrative coverage. The median coverage levels across the years were comparable, and did not show any significant change. The number of zero-dose children reached during SIAs was documented only in 19 SIAs conducted since 2018, of which 4 were reports from Nigeria, and 3 were from Ethiopia.

**Figure 2:**
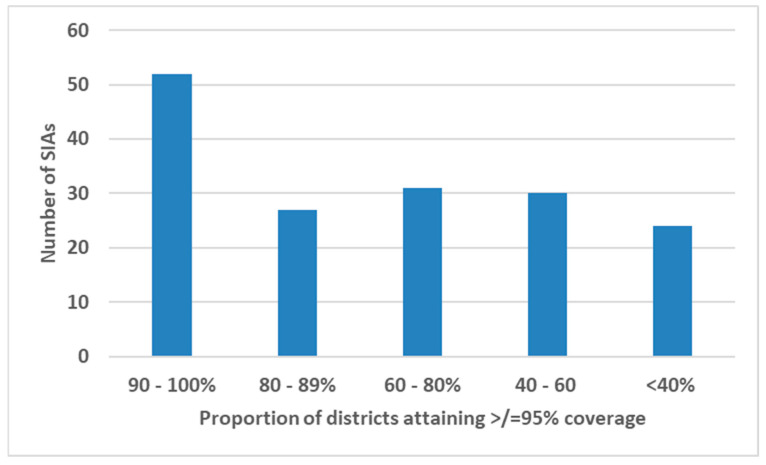
The number of measles/MR SIAs by the proportion of districts meeting the 95% administrative coverage targets in the African Region, 2001–2025.

Only 164 (51%) of the SIA reports provided data on the proportion of districts that have attained 95% administrative coverage. Of these, 11 SIAs (6.7%) had 100% of districts attaining 95% administrative coverage, while in 52 SIAs (32%) it was reported that 90% or more of districts reached 95% coverage. In 54 SIAs (33%), less than 60% of the districts attained 95% administrative coverage (Figure 2).

A post-campaign coverage survey (PCCS) was done following 94 of the 323 SIAs (29%). Of these, 18 (19%) had attained coverage of 95% or more, while 29 (31%) had coverage between 90% and 94.9%. National-level post-campaign survey coverage was reported to be less than 80% in 19 SIAs (20.4%). Administrative coverage at national level and PCCS coverage estimates had a difference of more than 20 percentage points in 22 SIAs (24%), while the difference was between 10 and 19.9 percentage points in 23 SIAs (25%), with survey coverage proving to be lower than administrative coverage. The biggest difference was documented in the SIAs in Central African Republic in 2013 and in South Sudan in 2014. In 15 SIAs (16%), the survey coverage was more than the reported administrative coverage at national level. All available post-campaign coverage survey results were more than the reported administrative coverage in Comoros, Eritrea, Guinea Bissau, Lesotho and Namibia.

### 3.3. Integrated Interventions in SIAs

In the SIA technical reports for the years 2006–2025, we reviewed available information about integrated interventions. It is noted that 169 (62%) of the 272 SIAs during the period had at least one additional intervention included with the measles/MR vaccination, while 61 SIAs (22%) clearly stated that no additional integrated intervention was implemented, and 42 reports (16%) did not have any information at all. Polio vaccination was integrated in 59 (35%) of these 169 measles/MR SIAs, while Vitamin A supplementation and deworming were integrated in 129 (76%) and 95 (56%) SIAs respectively. Ninety-one SIAs (54%) had integrated both Vitamin A supplementation and the distribution of deworming tablets. The distribution of insecticide-treated bed nets was integrated in 22 SIAs (13%), of which 18 were between 2006 and 2008. While the integration of Vitamin A and deworming is done nationwide in almost all SIAs, some of the integrated interventions like the distribution of bed nets and provision of additional campaign doses of vaccines (like TT, yellow fever or Meningitis A vaccines) took place in a subset of the measles/MR SIA target geographic areas.

The majority of SIA reports indicate that various activities have been undertaken to support routine immunization strengthening as part of SIA preparation and implementation, but the number of children vaccinated with other routine doses of vaccines was documented only during 17 measles/MR SIAs, as quantified in the technical reports, of which 14 were documented in the years after 2020. This includes the integrated implementation of Big Catch-up Vaccination in five countries in 2024 and 2025. Vaccination of adolescent girls against Human Papilloma Virus (HPV vaccination) was integrated in three SIAs.

According to the technical reports, Mauritania was the only country that has never included any additional intervention during the five nationwide preventive measles/MR SIAs conducted since 2004. On the other hand, some countries have integrated interventions not commonly encountered in immunization campaigns. Burundi included the administration of Praziquantel in three SIAs to prevent Bilharziasis. During the MR catch-up SIAs in 2014, Tanzania included the mass administration of ivermectin and albendazole for lymphatic filariasis control and the control of soil-transmitted helminthiasis respectively in 16 of the 27 target provinces. Nigeria integrated yellow fever and/or Meningitis A vaccination in multiple subnational measles SIAs between 2019 and 2024. In October 2025 the Nigeria measles–rubella catch-up SIAs included the provision of nOPV for children under five years, and also interventions against Neglected Tropical Diseases and malaria as follows: administration of oral Ivermectin (for children above 5 years of age) against Onchocerciasis in six LGAs in Kano State; administration of oral Ivermectin and Albendazole (for children above 5 years of age) against lymphatic filariasis in three LGAs in Oyo State; the administration of oral Azithromycin and Tetracycline ointment against Trachoma in three LGAs in Yobe State; and seasonal malaria chemoprophylaxis in Kano State. Ethiopia included the screening of specific disease conditions (along with referral for case management) in multiple measles campaigns between 2017 and 2025. These included the measurement of mid/upper-arm circumference as part of nutritional screening of children, as well screening for childhood clubfoot deformities, and for obstetric fistula among women.

## 4. Discussion

In the African Region, preventive SIAs constitute a major part of the measles elimination strategies, with a large number of measles-containing vaccine doses provided every year. The implementation of periodic SIAs remains a programmatic necessity in many countries, in order to close immunity gaps and avert large outbreaks. On average, 13 countries organize preventive SIAs every year, and 63 million doses of vaccine are provided annually. The lowest number of SIAs was in the year 2020 and corresponded to the COVID-19 pandemic which led to the postponement of many scheduled SIAs that year [10]. The implementation of time-sensitive and scheduled SIAs resumed in late 2020 starting with a few countries, with the introduction of social distancing and infection prevention and control measures [11]. The largest number of children vaccinated in SIAs was in 2025, with 18 countries conducting measles/measles–rubella preventive SIAs, including the subnational catch-up SIAs to introduce measles–rubella (MR) vaccine in DR Congo and in Nigeria.

The age targeting of measles/MR SIAs has been largely uniform with 85% of the follow-up SIAs targeting children up to 5 years of age. This is mainly dictated by the epidemiology of the confirmed measles cases and the need to protect the youngest birth cohorts who are most vulnerable to complications and deaths from measles. In addition, the limited availability of funding resources has influenced the upper age limit in SIAs. However, disease surveillance data from many countries shows that measles transmission often extends to older age groups [12,13,14,15]. The WHO measles vaccine position paper recommends that countries should triangulate surveillance data, vaccination coverage and survey data to determine the age distribution of susceptibility and hence the appropriate target age range for measles and MR campaigns [8].

Measles/MR SIAs have been vehicles for the integration of multiple interventions in numerous countries across the Region. Similar to findings documented in other studies, our data shows that Vitamin A supplementation was the most frequent health intervention to be integrated with measles SIAs [16,17]. On the other hand, very few countries included another injectable vaccine like yellow fever or Meningitis A vaccine for the same target population. Not all countries in the Region have epidemiological risks of yellow fever or epidemic meningitis. But in those countries at risk, the fact that only few countries implemented integrated multi-injection campaigns using these vaccines may be due to vaccine availability and programmatic considerations, the additional cold chain space demands, and the logistical complexities and concerns about vaccine safety while providing multiple injectables at the same time.

The success of integrated campaigns with the co-delivery of multiple interventions depends on the quality of joint planning and preparation as well as the timely availability of all needed resources and operational funding [18,19,20]. Integration of interventions has been shown to be effective in terms of reaching unreached populations using limited resources and allows disease control programs to attain equitable coverage [21,22,23,24].

We note that 33% of the SIAs reported administrative coverage of more than 100%, and there were major discrepancies between administrative and survey coverage results. Inaccuracies in the reporting of the number of doses administered, reliance on outdated census data and inaccurate denominators, and the vaccination of individuals outside the targeted age group or geographic area can all introduce errors in administrative reporting [25,26]. In this series, only 6.7% of SIAs managed to attain 95% administrative coverage in all districts, indicating the subnational coverage gaps, and sometimes the heterogeneity of coverage at subnational levels, even when the national-level data shows relatively high coverage. Similar administrative coverage gaps in SIAs have been reported in other WHO Regions as well. A data series from the Eastern Mediterranean Region covering the years 2013–2019 reported that only a third of 38 campaigns reached the 95% coverage target at national level, while 50% of 60 SIAs in the Western Pacific Region from the years 1970 to 2019 reached the 95% target [27,28]. However, unlike in the African Region, many of these countries have sustained high routine immunization coverage which has reduced the dependency on campaigns to close immunity gaps.

Conducting well-designed cluster surveys helps in generating more reliable coverage estimates at national and at the first subnational level, as recommended by the WHO [7,29]. Unfortunately, only a third of the SIAs in this study were followed by post-campaign coverage surveys. Various challenges have hampered the implementation of PCCS including inadequate funding, paucity of expertise in coverage survey planning and implementation, multiple and overlapping program priorities, and long delays in implementing surveys following the conclusion of SIAs [30,31].

In addition to tallying the total number of children reached during the SIAs, the WHO recommends monitoring the number and proportion of zero-dose children (children who never received any previous routine vaccination dose) who received their first dose of measles-containing vaccine during the SIAs [7]. Beyond administrative coverage figures, it is important to identify who is reached by SIAs to understand if the SIAs are achieving their objective [31,32]. Many of the SIA technical reports mention different targeted activities implemented to reach zero-dose children. However, only 19 SIA technical reports have documented the number of zero-dose children reached during the exercise. In recent years, post-campaign coverage surveys have systematically included the identification of zero-dose children as part of the data collected and reported.

In the last 25 years, the improvements in routine immunization coverage and the implementation of periodic SIAs have led to a general decline in measles and rubella cases across the Region [4]. In many countries, preventive SIAs have resulted in periods of low measles incidence. Even when resurgences occur, the level of transmission and sizes and durations of outbreaks are much lower than in the years before SIAs [33,34,35].

WHO guidance indicates that countries can consider stopping the conduct of SIAs once they attain and maintain very high measles vaccination coverage for the first and second doses [8]. Although few countries have reached this threshold, many countries have been widening the spacing of their inter-campaign intervals over the past decade, following sustained increases in routine immunization coverage and successful reduction in disease burden. For optimal impact, SIAs need to adequately reach unreached populations. This requires timely funding and adequate preparations among others. There is an urgent need for improved tools to identify unvaccinated children, high-risk populations, and innovative strategies to reach them, particularly amid declining global funding.

In order to fast-track progress towards the measles and rubella elimination goals, the African Regional program will need to apply the lessons, tools and technologies that were utilized by the global polio eradication program in various vaccination campaigns. These lessons and tools include the use of global champions and advocates, innovative approaches for social mobilization and communication, satellite mapping of communities, GPS tracking of vaccinators, and rapid and practical campaign evaluation approaches [36,37].

This study has some limitations. While every effort was made to source the data from the latest SIA technical reports, the reports may not be complete in tabulating the data from all interventions. The limitations of administrative coverage data have been very well described. In addition, national-level coverage figures often mask the gaps and heterogeneity of coverage at subnational levels. This manuscript focused on the quantitative aspect of SIA coverage and did not cover a qualitative review of lessons and best practices from campaigns, nor the impact of SIAs. Moreover, this analysis was limited to preventive SIAs intended to address immunity gaps and did not include data from outbreak response campaign activities.

## 5. Conclusions

Measles and MR SIAs have served as important platforms for the provision of multiple child survival interventions. However, there are documented gaps in vaccination coverage and in attaining homogeneously high coverage among districts. It is critical that SIAs reach children who have not benefited from routine doses, wherever they reside. Addressing these gaps in SIAs will require special efforts in demand generation, mapping of unreached communities, campaign logistics and resource coordination. A strong national leadership and ownership of the measles elimination strategies is a crucial ingredient for the attainment of the performance targets, including the targets for SIA coverage. All countries implementing SIAs should also include systematic monitoring of zero-dose children, and conduct post-campaign coverage surveys (PCCS) in a timely manner.

## Figures and Tables

**Table 1:** Annual measles/MR SIAs implemented and children reached, 2001–2025.

Year	Number of SIAs	Number of Persons Vaccinated	Median Administrative Coverage
2001	8	21,168,332	99.0%
2002	11	47,296,716	97.0%
2003	12	46,506,764	94.5%
2004	10	42,723,345	99.0%
2005	15	65,524,986	93.0%
2006	20	81,461,933	98.0%
2007	16	34,113,644	97.5%
2008	11	66,861,559	99.0%
2009	19	40,586,733	96.0%
2010	11	49,174,944	93.0%
2011	15	81,976,070	96.4%
2012	14	70,593,961	101.7%
2013	17	99,277,668	99.0%
2014	9	77,340,420	103.4%
2015	11	80,456,896	97.8%
2016	14	53,237,666	99.0%
2017	11	73,601,875	98.8%
2018	9	92,043,811	98.3%
2019	14	95,325,198	101.5%
2020	5	22,282,804	91.6%
2021	9	35,277,492	95.4%
2022	15	75,578,809	94.6%
2023	11	69,970,657	94.1%
2024	18	88,448,178	97.7%
2025	18	128,547,839	97.3%

## Data Availability

Dataset available on request from the authors.
